# Short-term exposure to particulate matter triggers a selective alteration of plasma extracellular vesicle-packaged miRNAs in a mouse model of multiple sclerosis

**DOI:** 10.3389/fimmu.2025.1596935

**Published:** 2025-07-03

**Authors:** Martino Bonato, Valentina Cerrato, Laura Dioni, Francesca Montarolo, Roberta Parolisi, Antonio Bertolotto, Valentina Bollati, Luca Ferrari, Enrica Boda

**Affiliations:** ^1^ Neuroscience Institute Cavalieri Ottolenghi (NICO), Department of Neuroscience Rita Levi-Montalcini, University of Turin, Turin, Italy; ^2^ EPIGET LAB, Department of Clinical Sciences and Community Health, University of Milan, Milan, Italy; ^3^ Neuroscience Institute Cavalieri Ottolenghi (NICO), Turin, Italy; ^4^ Koelliker Hospital, Turin, Italy

**Keywords:** air pollution, particulate matter, experimental autoimmune encephalomyelitis, multiple sclerosis, miRNA

## Abstract

Epidemiological studies have highlighted the existence of population groups exhibiting a higher sensitivity to the impact of environmental factors, such as exposure to air pollution. In these regards, people with Multiple Sclerosis (MS) or predisposed to develop MS - an autoimmune disorder of the Central Nervous System (CNS) - appear as a more vulnerable cohort to the effects of particulate matter (PM) exposure. Here, we aimed at disclosing the biological substrate of such higher vulnerability, and specifically at understanding whether individuals primed to develop autoimmunity (as it occurs in MS and in the experimental autoimmune encephalomyelitis - EAE - animal model of MS) respond differently to PM compared to healthy subjects. To this purpose, we characterized plasmatic extracellular vesicles (EVs) and their microRNA (miRNA) cargo in healthy and presymptomatic EAE mice early after exposure to PM_10_, compared to unexposed healthy and EAE mice. Results showed that the response of EAE mice to PM_10_ did not differ in terms of EV number or source, compared to that of healthy mice. Yet, remarkable differences existed in the identity of deregulated EV-associated miRNAs, which, in EAE mice, were predicted to target several MS-relevant biological processes and nervous system-, immune- and inflammation-related pathways, possibly contributing to disease worsening.

## Introduction

Despite increased public awareness and global improvements in air quality, air pollution remains a significant public health challenge for low-, middle-, and high-income countries. Particulate matter (PM) - a mix of solid particles and liquid droplets - is one of the main air pollutants, arising from natural sources (e.g. pollen, sea spray, and dust from ground erosion) and anthropic activities (e.g. combustion of fossil fuels, roadways and mining operations, agricultural activities; 1). Exposure to PM has been associated with an increased risk of cardiovascular and respiratory diseases, different types of cancer (2), and, more recently, pathologies affecting the central nervous system (CNS; 3–5).

PM includes coarse (PM_10_), fine (PM_2.5_), and ultrafine (PM_0.1_) particles - having an aerodynamic diameter smaller than 10, 2.5, and 0.1 μm, respectively - that typically comprise inorganic compounds, aromatic hydrocarbons, metals, and microbial components (6). When inhaled, PM penetrates the lungs and settles deep within, triggering a local inflammatory and defensive response that ultimately impacts all major organs and systems, including the CNS. (3, 4).

Although smaller particles - such as PM_0.1_ - could directly enter the blood circulation and overcome the blood-brain-barrier (BBB; 3), the disruption of the homeostasis of distant organs most likely relies on mechanisms other than a direct effect of PM particles. Alongside the secretion of proinflammatory cytokines, a well-established mechanism by which the effects of exposure to PM can extend beyond the respiratory compartment and trigger a systemic response, is the release of extracellular vesicles (EVs; 7–10). EVs are nano-sized (0.03–1 μm) plasma membrane fragments actively released by cells, which can be distributed to all systems, including the CNS (11) - *via* the blood and other biological fluids. EVs play a key role in the intercellular and inter-organ transfer of biological information. After reaching target cells through the recognition of surface-expressed ligands, EVs deliver their content, which can include proteins, lipids, metabolites, organelles, and genetic information. As regards this latter aspect, the most well studied molecules in EV cargo are microRNAs (miRNAs; 12), endogenous non-coding small RNAs, which play an important role in the post-transcriptional regulation of gene expression *via* either mRNA cleavage or translation inhibition. The power of miRNAs as regulatory mechanisms of cellular processes lies in their capability to tune the expression of multiple transcripts, exerting a broad control over functionally related target mRNAs. Moreover, different miRNAs can act together to cooperatively target mRNAs that encode proteins within the same functional network (13). Thus, deregulation of even a restricted set of miRNAs can significantly impact gene expression, and - as a consequence - EVs are able to profoundly influence the molecular state and function of target cells (14). Consistently, shuttling of miRNAs packaged in plasmatic EVs has emerged as one of the most powerful mechanisms linking PM exposure with the disruption of homeostasis in extrapulmonary compartments (7, 15, 16).

Epidemiological studies have strongly indicated that environmental factors play a role in shaping the geographical distribution and the timing of hospital admissions for a number of diseases. In individuals with a predisposing background, environmental factors have been proposed to “set the disease threshold” (17). Specifically, as regards PM, while most individuals can maintain homeostasis even upon chronic or high dosage exposure, certain vulnerable groups exhibit heightened sensitivity to PM impact (18). This increased susceptibility is attributed to genetic or acquired factors that make the biological responses to PM exposure differ from those elicited in “coping” individuals (18).

Lifestyle and environmental factors (e.g. smoking, limited sun exposure, low vitamin D, obesity, infections and exposure to environmental pollutants) have been associated with increased risk of Multiple Sclerosis (MS; 19–21). In particular, airborne PM peaks have been associated with higher rates of hospital admissions for MS onset or relapses (22–30), suggesting that people with MS (or predisposed to develop MS) are a PM-vulnerable cohort. MS is a chronic disease with autoimmune components, characterized by immune cell infiltration in the CNS, neuroinflammation, diffuse myelin loss, and, over time, neurodegeneration. These processes result in functional impairments and, in many cases, cognitive and psychiatric symptoms (31–33). Of note, PM exposure has been reported to trigger MS-relevant events (e.g. inflammation, endothelial and BBB alterations, CNS white matter damage, and changes in brain innate immune cell activity) even in healthy individuals (34, 35). This response seems to be more pronounced in MS patients (29). Yet, the biological substrate of such vulnerability to PM remains uninvestigated so far.

Here, we aimed at understanding whether individuals primed to develop autoimmunity against CNS myelin (as it occurs in MS and in the MOG_35-55_-induced experimental autoimmune encephalomyelitis - EAE - animal model of MS) respond differently to PM compared to healthy subjects. Specifically, we characterized EVs (i.e. number, size and cellular source) and EV-associated miRNA profile in the plasma of healthy (Ctrl) and presymptomatic EAE mice acutely exposed to PM_10_, compared to unexposed Ctrl and EAE mice. These analyses showed that the response of mice primed to develop EAE to PM exposure differed from that of healthy Ctrl mice and did not simply correspond to the amplification of differences already existing between EAE and healthy mice. Although the miRNA cargo of plasma EVs released following PM exposure had a signature relevant for MS in both healthy and EAE mice, deregulated miRNAs were distinct and the response of EAE mice appeared more “pathogenic” than that of healthy mice. *In silico* analyses were applied to identify target gene transcripts and biological processes possibly underlying PM effects, unveiling a significant enrichment in nervous system-, immune- and inflammation- related pathways. Overall, our data support the idea of a synergy between PM exposure and immune system priming toward autoimmunity, and suggest that shuttling of EV-associated miRNAs in response to PM exposure may participate in different aspects of MS pathogenesis and exacerbation.

## Materials and methods

### Animals and experimental design

Mice were housed in the vivarium under standard conditions (12-hr light/12-hr dark cycle at 21°C) with food and water *ad libitum*. The project was designed according to the guidelines of the NIH, the European Communities Council (2010/63/EU) and the Italian Law for Care and Use of Experimental Animals (DL26/2014). It was also approved by the Italian Ministry of Health (authorization 510/2020-PR to EB) and the Bioethical Committee of the University of Turin. The study was conducted according to the ARRIVE guidelines.

### Chronic EAE induction

To induce chronic EAE, 8 week-old female C57BL/6J mice (Charles River, Calco, Italy) were immunized by 2 subcutaneous injections of 200 μg myelin oligodendrocyte glycoprotein 35–55 peptide (MOG_35-55_; Espikem, Florence, Italy) in incomplete Freund’s adjuvant (IFA; Sigma-Aldrich, Milan, Italy) containing 8 mg/ml Mycobacterium tuberculosis (strain H37Ra; Difco Laboratories Inc., Franklin Lakes, NJ, USA), followed by 2 intravenous injections of 500 ng of Pertussis toxin (Duotech, Milan, Italy) on the immunization day and 48 h later (36).

### PM_10_ administration and blood collection

We exposed healthy (Ctrl) and EAE mice to a commercially available urban outdoor PM_10_ (NIST Standard Reference Material SRM1648a; Sigma-Aldrich), which is a chemically standardized compound (https://tsapps.nist.gov/srmext/certificates/1648a.pdf), widely used in toxicological studies (37–39). Stock suspensions of NIST SRM1648 PM were prepared in sterile ultrapure water and aliquots were thoroughly mixed under sonication for 1 hour prior to each experiment. Mice were randomly divided into control (saline, i.e. NaCl 0.9% solution) and treatment (PM) groups. The treatment group was treated by intratracheal instillations (Intubation stand, Kent Scientific Corporation) of a PM_10_ suspension (10 μg in 50 μl of saline), and the control group was treated with saline (50 μl), as in our previous study (40). We opted for the intratracheal administration of PM to assure low variability of absorption and exclude a direct nose-to-CNS transit of PM via the olfactory mucosa.

PM_10_ dose was calculated considering the daily respiratory volume of mice (0.04 m^3^) and the daily peaks of PM_10_ concentration in East Europe polluted areas (>250 μg/m^3^; 41). Therefore, in order to use a PM_10_ dose relevant for human exposure, the dose used in this study was set as 10 μg (0.04 m^3^/day × 250 μg/m^3^). PM_10_ was administered 4 days after EAE immunization, i.e. during the presymptomatic phase of the EAE disease course, where debilitating symptoms are yet to be displayed by the animals (i.e. clinical score=0, according to the conventional EAE clinical score assessment (36)). Peripheral blood of each mouse was collected in EDTA Vacutainer tubes (Becton Dickinson, New Jersey, USA), 6 hours after PM_10_/saline exposure, and processed within 3 hours from sampling. Blood was centrifuged at 1300xg for 15 min to separate plasma.

### EVs isolation and characterization

For EV studies, we followed the MISEV2018 guidelines (42; **Supplementary Table S1**, **Supplementary Figure S1A, B**). EVs have been isolated from plasma pools from six mice of each condition: healthy mice which received saline (Ctrl); healthy mice which received PM_10_ (Ctrl PM); EAE mice which received saline (EAE); EAE mice which received PM_10_ (EAE PM). The resulting pooled samples were centrifuged three times at increasing speeds (1000xg, 2000xg, 3000xg) for 15 min at 4°C. After every centrifugation, the supernatant was decanted into a new tube and centrifuged again to remove cell debris and aggregates. Shortly after centrifugation, plasma samples were transferred into ultracentrifuge tubes (Polycarbonate Centrifuge Bottles, Beckman Coulter) and filled up to 10.5 ml with NaCl 0.9% solution. Ultracentrifugation was performed at 110,000xg for 75 min at 4°C to allow sedimentation of EVs. After ultracentrifugation, the supernatant was discarded and tubes were allowed to air dry for 2 min.

To confirm that this isolation protocol resulted in preparations enriched in EVs, western blotting (WB) was used to assess the expression of EV markers (i.e. the tetraspanins CD63/CD9) and absence of contamination from other cellular components (i.e. calnexin, a marker for endoplasmic reticulum; **Supplementary Figure S1A**). Briefly, plasma EVs lysates were obtained in RIPA buffer (150 mM NaCl, 1% Triton X-100, 0.5% Sodium deoxycholate, 0.1% sodium dodecyl sulfate, 50 mM Tris Base, 5mM EDTA, 1mM EGTA) containing protease and phosphatase inhibitors (100mM DTT, 1M NaF, 200 mM sodium orthovanadate, 100 mM PMSF), boiled in SDS buffer and separated using a 10% SDS-PAGE gel. Proteins were transferred to PVDF membranes, blocked in 5% non-fat milk in TBS (20 mM Tris-HCl, pH 7.5, 150 mM NaCl) + 0.1% Tween-20 (TBS-T) for 1h and then incubated with primary antibodies (anti-rabbit CD63, 1:500, Immunological Sciences, Italy; anti-mouse CD9, 1:250, BD Biosciences; anti-rabbit calnexin 1:250, Cell signaling, USA) overnight at 4°C. After washes with TBS-T, membranes were incubated with appropriate secondary antibodies (anti-rabbit or anti-mouse, 1:3000, Sigma, Italy) for 1h at room temperature. Protein extract from a mouse cerebral cortex was used as a positive control. The chemiluminescent signal was visualized using Westar Antares ECL Blotting Substrates (Cyanagen; Italy), acquired with Bio-Rad ChemiDocTM Imagers (Bio-Rad; Italy).

Nanoparticle tracking analysis (NTA) and flow cytometry were used to characterize EV number/size (**Supplementary Figure S1B**) and cellular source, respectively (as in 43). To determine EV cellular origins, immunophenotyping was achieved with the MACSQuant Analyser flow cytometer (Miltenyi Biotec, Bergisch Gladbach, DE) following the manufacturer’s protocol. The Fluoresbrite Carboxylate Size Range Kit I (0.2, 0.5, 0.75, and 1 µm) was used to set the calibration gate on the MACSQuant Analyser system. To evaluate the integrity and to highlight EV subsets, 60-µL sample aliquots were stained with 0.02 µM 5(6)-carboxyfluorescein diacetate *N*-succinimidyl ester (CFSE) at 37°C for 20 min in the dark. CFSE is a vital non-fluorescent dye that enters EVs, where intracellular esterase enzymes remove the acetate group and convert the molecule into the fluorescent ester form. To characterize and count EVs, the following panel of antibodies was used: monoclonal APC-anti-CD14 (Clone REA934; dilution 1:10) to identify EVs from macrophages and/or monocytes; APC-antiCD41 (Clone: REA1194; dilution 1:10) for EVs from platelets; APC-anti-CD25 (clone: REA568; dilution 1:10) for EVs from regulatory T (Treg) lymphocytes. All antibodies were purchased from Miltenyi Biotec. Quantitative multiparameter analysis of flow cytometry data was conducted using FlowJo software (Tree Star, Inc., Ashland, OR, USA) and antibody gating strategies were performed as previously described (43).

### EV-miRNAs isolation

miRNA isolation from EV pellets in tubes was performed using the miRNeasy Mini Kit (Qiagen, Venlo, NL) according to the manufacturer’s protocol, with the addition of the “Rneasy Mini Elute Clean-up” kit (Qiagen). To assess the quality of miRNA purification, samples were analyzed by 2100 Bioanalyzer (Agilent Technologies, Santa Clara, CA, USA) using Agilent RNA 6000 Pico Kit (**Supplementary Figure S2**) and stored at -80°C.

### miRNA profiling

Reverse Transcriptase reaction was performed using Megaplex™ RT Primers Pool A and B Rodent (Life Technologies, Foster City, CA) and TaqMan**^®^** MicroRNA Reverse Transcriptase Kit (Life Technologies). Briefly, two distinct reactions (A and B) were performed, to cover the reverse transcription of 758 target miRNAs, including 0.75 µl of Megaplex RT Pool, 0.15 µl of dNTPs (100 mM), 0.75 µl 10X RT Buffer, 0.90 µl of MgCl_2_ (25 mM), 0.1 µl of RNases Inhibitor (20 U/µl) and 1.5 µl of MultiScribe™ Reverse Transcriptase (50 U/µl). The reverse transcription thermal protocol consisted of 40 cycles at 16°C for 2 min, 42°C for 1 min, and 50°C for 1 s, plus one cycle at 85°C for 5 min and final stage at 4°C. The cDNA samples were pre-amplified using “Low Sample Input Protocol (LSI) for profiling human miRNA using OpenArray^®^ Platform” (Application Note 2011- Life Technologies) with a specific Megaplex™ Preamp Primer Mix for Pool A and Pool B (Rodent). The pre-amplified samples were diluted 1:20 with nuclease-free water and were analyzed by QuantStudio™ 12K Flex Real-Time PCR System with OpenArray^®^ Platform (Applied Biosystems) according to manufacturer’s instructions.

### miRNA expression data analysis

The Gene Expression Suite Software was used to analyze the expression of 754 unique miRNAs. Four small RNA species (RNU48, RNU44, U6, and ath-miR159a), included in the array as internal technical controls to assess assay performance and RNA integrity, were also present but not used for normalization purposes. Only miRNAs with Relative Threshold Cycle (C_rt_) value< 27 or AmpScore> 1.24 were considered amplified. The expression data were normalized using the “Global Normalization Factor” which calculates the average Crt of all detected miRNAs in a given sample. This method is recommended for EV-derived RNA, where validated endogenous reference genes are lacking due to the heterogeneity of vesicle content. Relative miRNA expression (also referred as Fold Change - FC) was determined using the relative quantification RQ= 2^(-Δcrt)^ formula, with -ΔC_rt_ = measured C_rt_ -medium normalization C_rt_ (44).

### Statistical analyses

**Figure 1:**
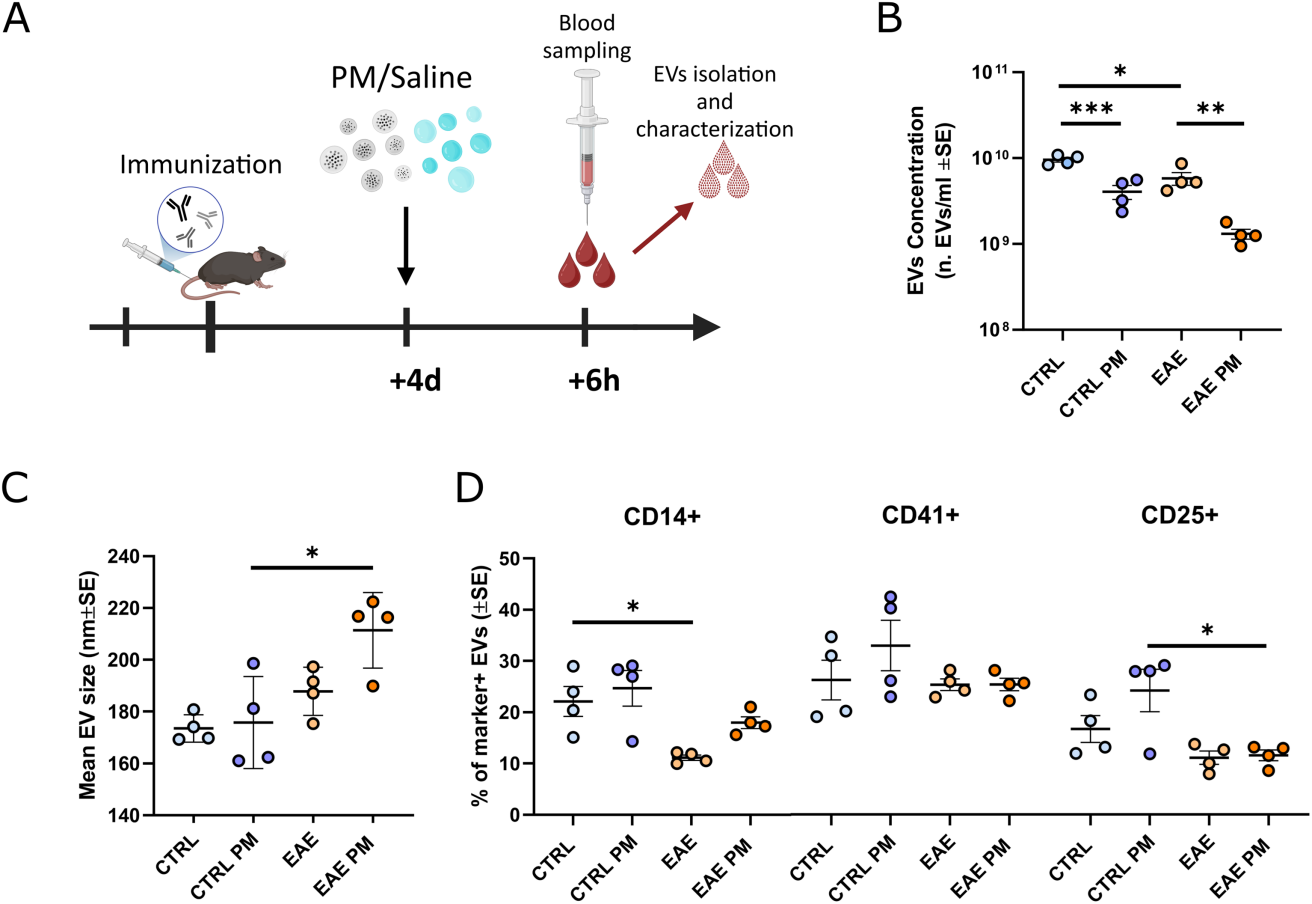
Characterization of mouse plasmatic EVs isolated after an acute PM_10_ exposure. **(A)** Schematic representation of the experimental design (graphics created with BioRender.com). **(B, C)** Mean EVs concentration **(B)** and size **(C)** in mouse plasma of CTRL and EAE mice exposed either to saline or PM_10_. **(D)** Percentage (%) of EVs positive for markers of monocytes/macrophages (CD14+), platelets (CD41+) and T-regs (CD25+). Each dot represents an individual mouse. Differences were assessed by Two-way ANOVA (see **Supplementary Table S2** for P and F values of each comparison). *p<0.05; **p<0.01; ***p<0.001.

Statistical analyses were carried out with GraphPad Prism 9 (GraphPad software, Inc, RRID: SCR_002798). As for EV characterization (i.e. EV concentration, mean EV size and percentage of marker-positive EVs; **Figure 1**), the Shapiro-Wilk test was first applied to test for a normal distribution of the data. Then, as data were normally distributed, a Two-ways ANOVA test followed by Bonferroni’s *post-hoc* analysis was used for multiple group comparisons. P*<*0.05 was considered as statistically significant. Statistical differences were indicated with * P*<*0.05, **P*<*0.01, ***P*<*0.001, ****P*<*0.0001. The list of the applied tests and number of animals in each case are included in **Supplementary Table S2**. Differential expression analysis of 754 unique miRNAs between groups was assessed using unpaired t-tests followed by the Benjamini-Hochberg FDR correction for multiple testing. A threshold of 0.20 was applied to the FDR p-value significance level to identify the set of top miRNAs.

### Network analysis of deregulated miRNAs and KEGG pathway enrichment analysis

The interactions between deregulated miRNAs and their target gene transcripts in each experimental condition were visualized using the web-based platform miRNet 2.0 (https://www.mirnet.ca/miRNet/home.xhtml 45). Significantly upregulated or downregulated miRNAs from each of the four groups, identified by their miRBase IDs, were uploaded into the “miRNA” module of miRNet 2.0. The target list was specified as “Genes (miRTarBase v9.0)” to link the input miRNAs to their predicted target gene transcripts, generating interaction tables and network visualizations. In the resulting networks, miRNAs may appear as specific strands (e.g., miR-9-5p from the input miR-9) or as closely related variants (e.g., miR-451a from miR-451). For the sake of clarity, only the most highly connected miRNAs in each network were labeled in the visualizations. KEGG pathway enrichment analysis was performed on the target gene lists from each network using the “Function Explorer” toolbox within miRNet, which employed a hypergeometric test to identify enriched (p-value < 0.05) pathways by comparing the input genes against the full set of mouse genes annotated in the KEGG database. The resulting p-values were adjusted for multiple testing using the Benjamini-Hochberg procedure to control the false discovery rate (FDR).

### Gene-to-disease association analysis

The list of target genes identified by the miRNA network analysis was used as input for a gene-to-disease association analysis using the DisGeNET Curated dataset. Genes associated with a disease class were displayed using the DisGeNET application in Cytoscape (46), operated through a DisGeNET specific R script. Edge thickness in the network graphical representation is proportional to the DisGeNET score for association robustness (S_GDA_, score of Gene to Disease Association), which takes into consideration the number and type of sources and number of publications that support the association, following the subsequent formula:

S_GDA_=C+M+I+L+T
C = score for number of curated sources supporting the GDA (range from 0 to 0.7)
M = score for number of model sources supporting the GDA (range from 0 to 0.1)
I = score for number of inferred sources supporting the GDA (range from 0 to 0.05)
L = score for number of publications supporting the GDA (range from 0 to 0.4)
T = score for number of clinical trials supporting the GDA (range from 0 to 0.1). Further information can be found at https://disgenet.com/About#metrics.

## Results

### Acute exposure to PM_10_ similarly affects circulating EV release in healthy and presymptomatic EAE mice

To assess whether individuals primed toward the development of autoimmunity against CNS myelin (i.e. immunized to develop EAE) react differently to PM_10_ exposure, we characterized plasmatic EVs (number/size, source and miRNAs cargo) in healthy (Ctrl) *vs.* presymptomatic (i.e. 4 days after immunization) EAE mice exposed to PM_10_, compared to Ctrl and presymptomatic EAE mice which received saline (**Figure 1A**). Although not resulting in an overt motor impairment, nor in demyelination, in the presymptomatic stage of EAE the biological processes leading to the pathology have already started, as exemplified by cytokine and chemokine alterations in the nervous tissue, cerebrospinal fluid (CSF), and plasma (47), changes in the expression levels of molecules involved in neuronal-microglial communication (48), as well as by mouse cognitive impairment (49), thus recapitulating the early stages of MS.

EAE mice basally showed a slightly lower concentration of plasmatic EVs compared to Ctrl. PM_10_ exposure induced a further significant decrease in plasmatic EV release in both Ctrl and EAE mice, compared to unexposed individuals (**Figure 1B**). Although reduced, about 1.2 x10^9^ EVs/ml were still found in the plasma of PM_10_-exposed EAE mice (**Figure 1B**), showing a moderate increase in size, compared to those of unexposed EAE and PM_10_-exposed and unexposed Ctrl mice (**Figure 1C**). To characterize EV cellular source, we exploited a panel of markers specific for cell types reported to be at the origin of plasma EVs upon PM exposure in humans, i.e. monocytes/macrophages and platelets (8, 15), and important players in MS pathophysiology, i.e. T regulatory cells (Tregs; 50). While in Ctrl mice 20-25% of EVs were produced by monocytes/macrophages and by Tregs, only 10% of EVs have these cellular origins in EAE mice, as assessed by immunopositivity for CD14 and CD25 surface antigens respectively (**Figure 1D**). Although the fraction of monocyte/macrophage-derived EVs appeared slightly higher in PM_10_-exposed EAE mice compared to unexposed EAE, overall PM_10_ exposure did not alter the percentage of marker-positive EVs in both Ctrl and EAE mice (**Figure 1D**).

Taken together, these analyses showed that – in terms of plasmatic EV number and source - mice primed to develop autoimmunity do not respond differently to PM_10_ exposure, compared to healthy Ctrl mice.

### Selective deregulation of extracellular vesicle-packaged miRNAs in EAE mice after short-term exposure to PM_10_

**Figure 2:**
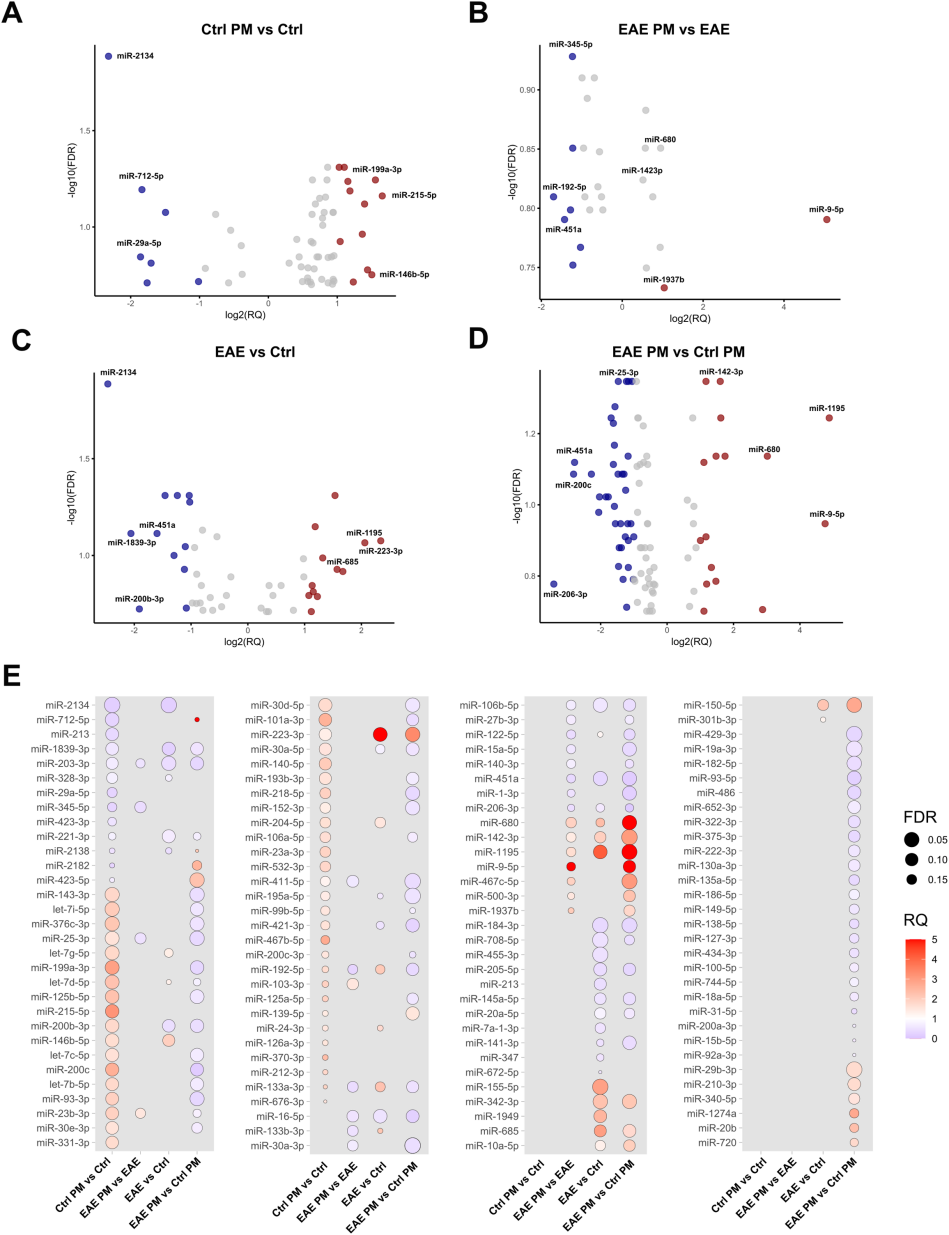
Deregulated EV-packaged miRNAs. Volcano Plots showing upregulated and downregulated miRNAs (p<0.05; FDR<0.2) in **(A)** Ctrl PM_10_ vs. Ctrl, **(B)** EAE PM_10_ vs. EAE, **(C)** EAE vs. Ctrl and **(D)** EAE PM_10_ vs. Ctrl PM_10_ mice. Top deregulated miRNAs are highlighted in red (upregulated, RQ >1) and blue (downregulated, RQ<0.5). **(E)** Dot plots showing differentially expressed miRNAs across comparisons, with dot size indicating FDR and color representing RQ. See **Supplementary Table S3** for the entire list of deregulated miRNAs.

To assess whether the response to PM_10_ of presymptomatic EAE mice differed in terms of EV miRNA cargo, miRNAs have been extracted and quantified from plasmatic EVs. This analysis provided a list of deregulated miRNAs for each of the following comparisons: PM-exposed Ctrl (i.e. Ctrl PM) vs. unexposed Ctrl (i.e. Ctrl); unexposed EAE (i.e. EAE) vs. unexposed Ctrl mice; PM-exposed EAE (i.e. EAE PM) vs. unexposed EAE; PM-exposed EAE vs. PM-exposed Ctrl mice (**Figure 2**). Deregulated miRNAs are listed in **Supplementary Table S3**. In Ctrl mice, PM_10_ exposure induced changes in the expression of 59 miRNAs, among which the most downregulated ones were miR-2134, miR-712-5p and miR-29a-5p, while the most upregulated were miR-215-5p, miR-199a-3p and miR-146b-5p (7 miRNA species with FDR<0.05; **Figures 2A, E**). Of note, miR-2134 was also one of the most downregulated miRNAs among the 45 differentially expressed miRNAs in EAE *vs.* Ctrl mice, together with miR-1839-3p, miR-200b-3p and miR-451a. Top upregulated miRNAs in EAE *vs.* Ctrl mice instead included miR-223-3p, miR-1195 and miR-685 (6 miRNA species with FDR<0.05; **Figures 2C, E**).

**Figure 3:**
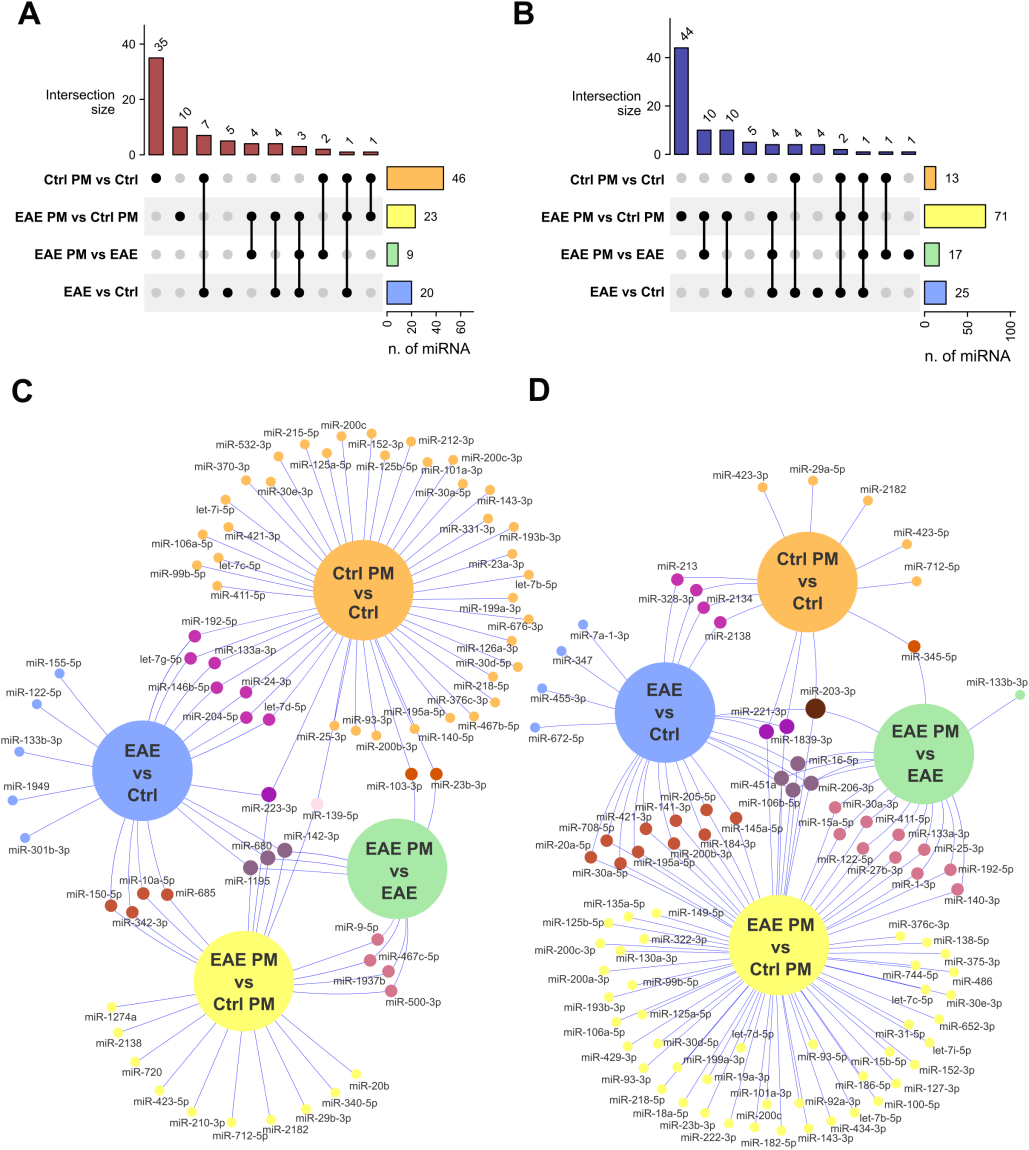
Overlap of deregulated EV-associated miRNAs among groups. **(A, B)** Upset plots showing the number of upregulated **(A)** and downregulated **(B)** miRNAs that are selectively or commonly expressed among comparisons. **(C, D)** Venn networks showing the identity of miRNAs upregulated **(C)** or downregulated **(D)** in all comparisons of interest. See **Supplementary Table S4** for the entire list of deregulated miRNAs that are selectively or commonly expressed among comparisons.

When comparing EAE mice exposed to PM_10_ to unexposed EAE mice, we observed 26 differentially expressed miRNAs (**Figures 2B, E**). In particular, miR-192-5p, miR-451a and miR-122-5p were the most downregulated, whereas miR-9-5p and miR-680 were the most up-regulated. Finally, 94 miRNAs were differentially expressed between EAE and healthy (Ctrl) mice exposed to PM_10_, with miR-200c, miR-451a and miR-25-3p among the most downregulated, and miR-9-5p, miR-1195 and miR142-3p among the most upregulated miRNAs (7 miRNA species with FDR<0.05; **Figures 3D, E**).

Notably, a certain degree of overlap existed between the responses of healthy mice to immunization and to PM_10_, especially in terms of downregulated miRNAs (58.84% of the miRNAs downregulated in Ctrl PM vs. Ctrl mice overlapped with those downregulated in EAE vs. Ctrl mice; **Figure 3**; **Supplementary Table S4**), suggesting a similar biological effect. In contrast, the responses to PM_10_ of EAE and healthy mice were largely divergent, with only 22% of upregulated miRNAs and 12% of miRNAs downregulated in EAE PM vs. EAE overlapping with those downregulated in Ctrl PM vs. Ctrl mice (**Figure 3**; **Supplementary Table S4**).

Overall, these analyses show that the response to PM_10_ – in terms of deregulated EV-associated miRNAs – is qualitatively different in mice primed to develop autoimmunity compared to healthy Ctrl mice.

### miRNA target prediction and network analysis

**Figure 4:**
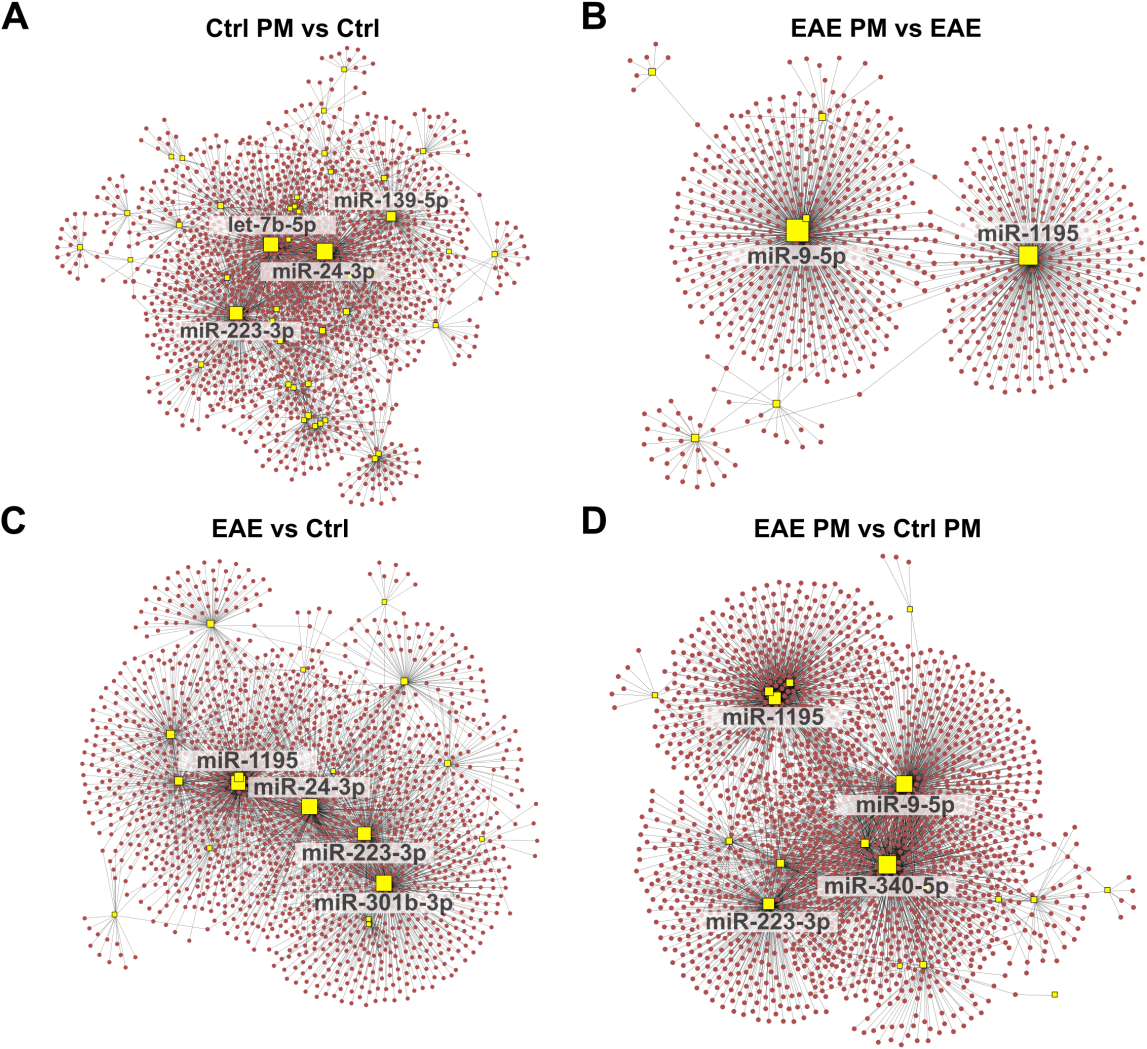
Network analysis of upregulated miRNAs and their predicted target gene transcripts. Network diagrams represent the interactions between upregulated miRNAs and their predicted target gene transcripts in the following conditions: **(A)** Ctrl PM_10_ *vs* Ctrl, **(B)** EAE PM_10_ *vs* EAE, **(C)** EAE vs Ctrl, and **(D)** EAE PM_10_ *vs* Ctrl PM_10_. Yellow squares indicate miRNAs, and red dots represent target gene transcripts. For clarity, only the names of miRNAs with more connections (threshold varies across networks) are labeled. See **Supplementary Table S5** for the entire list of upregulated miRNAs and their predicted target gene transcripts.

**Figure 5:**
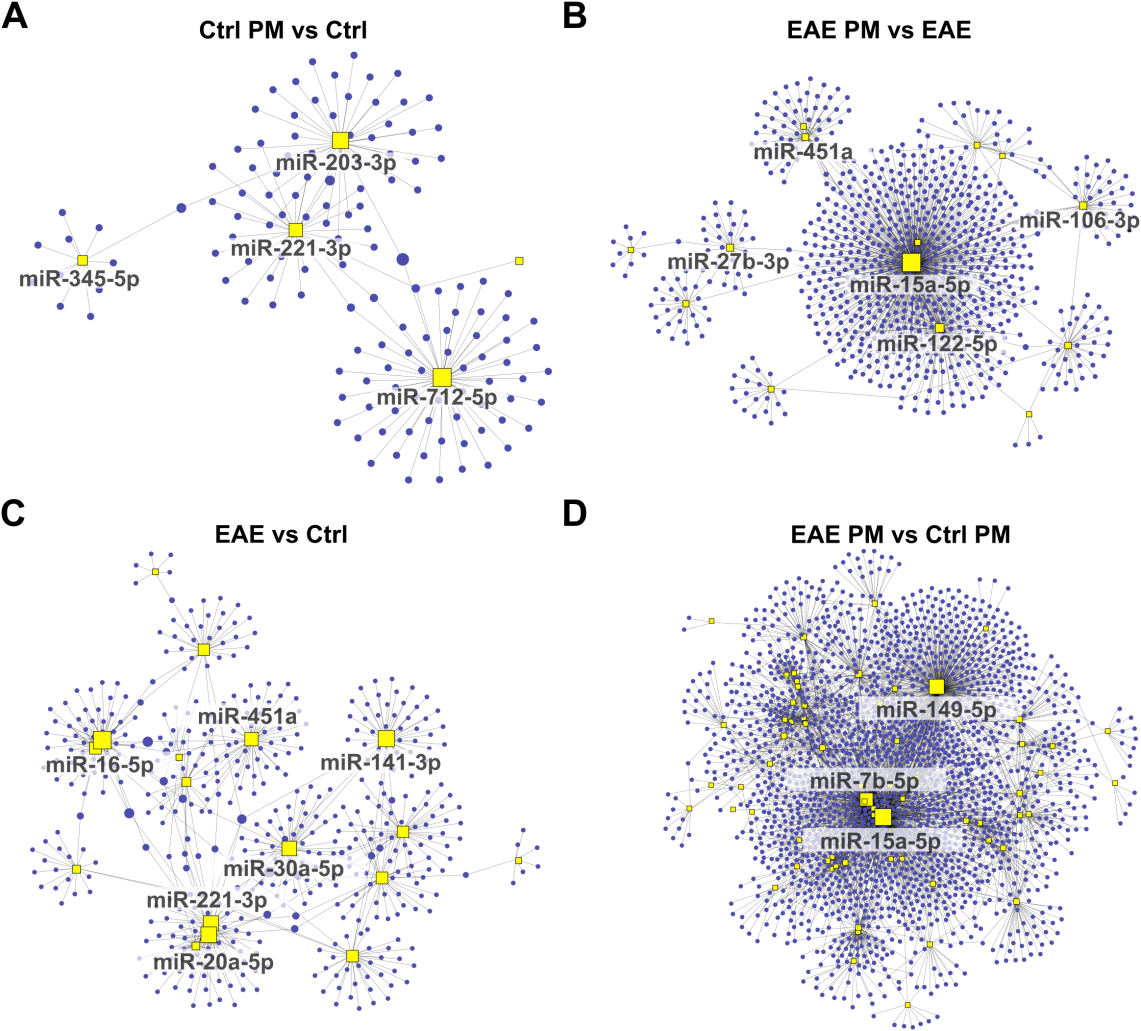
Network analysis of downregulated miRNAs and their predicted target gene transcripts. Network diagrams represent the interactions between downregulated miRNAs and their predicted target gene transcripts in the following conditions: **(A)** Ctrl PM_10_ *vs* Ctrl, **(B)** EAE PM_10_ *vs* EAE, **(C)** EAE vs Ctrl, and **(D)** EAE PM_10_ *vs* Ctrl PM_10_. Yellow squares indicate miRNAs, and blue dots represent target gene transcripts. For clarity, only the names of miRNAs with more connections (threshold varies across networks) are labeled. See **Supplementary Table S5** for the entire list of downregulated miRNAs and their predicted target gene transcripts.

To further explore the potential functional impact of miRNA deregulations triggered by PM_10_ in healthy and EAE mice, we identified the target gene transcripts of miRNAs that were differentially expressed in the 4 comparisons (**Supplementary Table S5**). Notably, several targets were shared among multiple deregulated miRNAs, suggesting potential regulatory hubs. In line with this idea, network-based analyses performed on upregulated (**Figure 4**) and downregulated (**Figure 5**) miRNAs for each experimental comparison highlighted a significant number of interactions between the deregulated miRNAs (yellow squares) and their predicted target gene transcripts (red dots for upregulated miRNAs in **Figure 4** and blue dots for downregulated miRNAs in **Figure 5**). Overall, the networks of upregulated miRNAs (**Figure 4**) were remarkably more complex than those of the downregulated miRNAs (**Figure 5**), which revealed relatively few target gene connections (especially for Ctrl PM *vs* Ctrl and EAE *vs* Ctrl, with fewer than 50 interactions; **Figures 5A, C**). In contrast, the networks of downregulated miRNAs in EAE PM *vs* both EAE (**Figure 5B**) and Ctrl PM (**Figure 5D**) exhibited more intricate interactions, with a few miRNAs having over 300 connections each.

Examining the shared miRNAs within these networks allowed us to provide more insightful information about the actual biological consequences of miRNA deregulation, accounting for both the miRNA-target gene transcript interactions and their collective regulatory impact, rather than simply considering the shared miRNAs across conditions. Interestingly, among the upregulated miRNAs displaying the highest number of connections, a partial overlap appeared again between the response of healthy mice to immunization (EAE *vs* Ctrl) and to PM_10_ (Ctrl PM *vs* Ctrl; e.g. miR-24-3p, and miR-223-3p among the upregulated miRNAs and miR-221-3p among the downregulated miRNAs; **Figures 4A, C**, **5A, C**), suggesting that these miRNAs may lead to similar cascades in response to both environmental and autoimmune triggers. Except for miR-1195, miR223-3p and miR-451a which were shared across EAE *vs* Ctrl and EAE PM *vs* Ctrl PM (**Figures 4B–D**), “hub” miRNAs deregulated in EAE mice responding to PM_10_ were exclusive. Among them, miR-9-5p (**Figures 4B, D**) and miR-15a-5p (**Figures 5B, D**) were the most connected upregulated and downregulated miRNAs, respectively.

**Figure 6:**
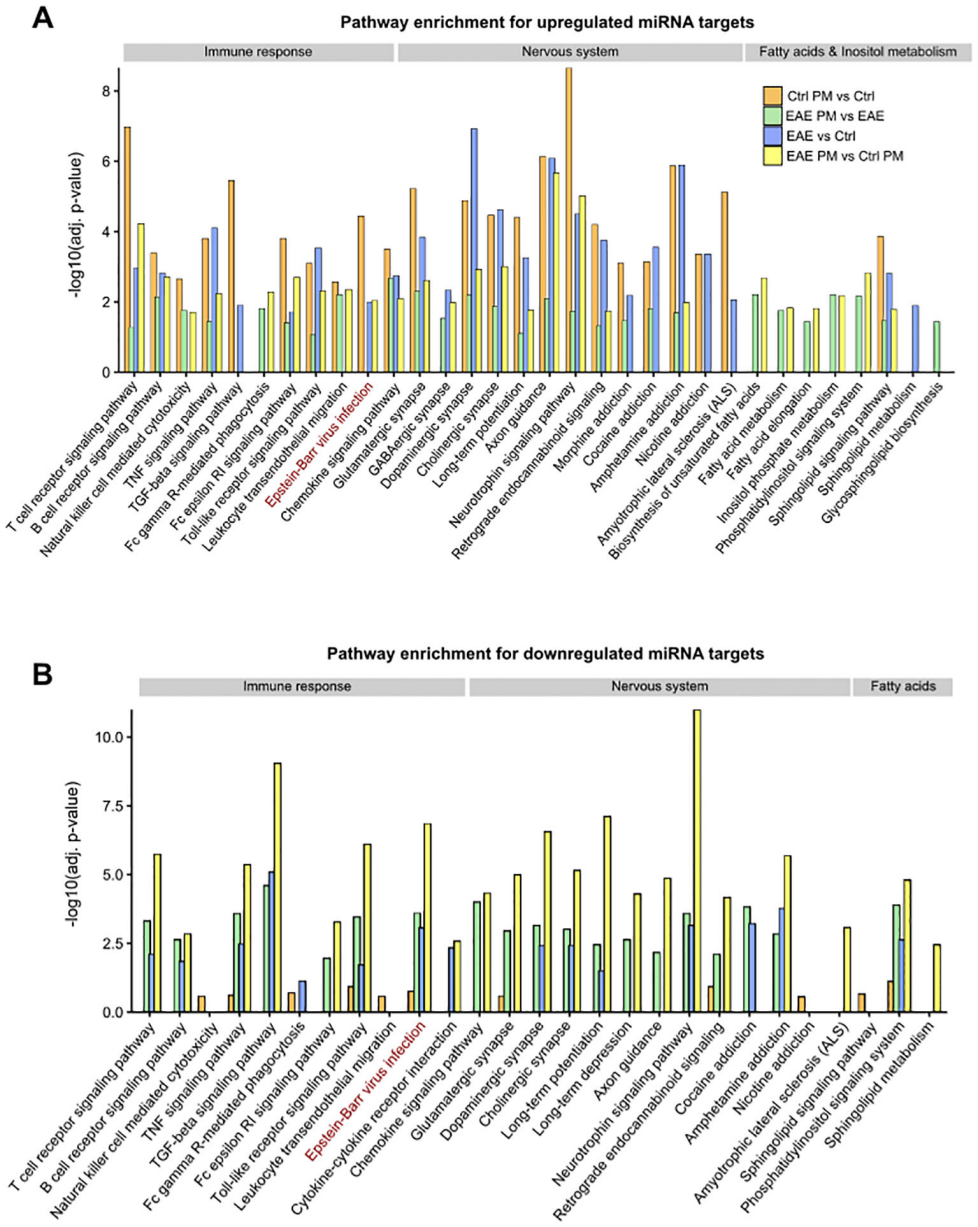
KEGG Pathway enrichment analysis of miRNA target networks. Bar plots showing selected significantly enriched pathways (adjusted p < 0.05) for the target genes of upregulated **(A)** and downregulated **(B)** miRNAs across the distinct comparisons. The y-axis indicates the –log_10_ of the adjusted p-values (adj. p-value). The displayed pathways represent those most relevant to nervous system function, immune response, and fatty acid/inositol phosphate metabolism. See **Supplementary Table S6** for the entire list of enriched pathways.

To further investigate the biological pathways potentially influenced by the detected miRNA deregulation, KEGG pathway enrichment analysis was performed on the predicted target genes from the upregulated and downregulated miRNA networks across the different comparisons. Of relevance in the context of an autoimmune CNS disease like MS, this analysis revealed a significant enrichment of terms related to synapse function, nervous system, and immune response, for both up- and downregulated miRNAs (**Figures 6A, B**, respectively, **Supplementary Table S6**). Terms such as *Glutamatergic synapse*, *GABAergic synapse*, *Dopaminergic synapse*, *Cholinergic synapse* suggest that miRNA deregulation may play a role in modulating synaptic activity and plasticity, as further supported by the enrichment of *Long-term potentiation* and *Long-term depression* pathways. Moreover, several terms associated with addiction - such as *Morphine addiction*, *Cocaine addiction*, *Amphetamine addiction*, and *Nicotine addiction* - emerged across the comparisons, suggesting a possible influence of deregulated miRNAs on neurotransmitter systems. Besides nervous system-related pathways, immune- and inflammation-related pathways were also significantly enriched. These pathways were significantly enriched in the networks of upregulated miRNAs across the different comparisons (**Figure 6A**; **Supplementary Table S5**). In contrast, for downregulated miRNAs, nervous system- and immune-related pathways emerge almost exclusively in the comparisons involving EAE mice (i.e. EAE *vs* Ctrl, EAE PM *vs* Ctrl PM and EAE PM *vs* EAE comparisons). Although this might be due only to the very small size of the network of downregulated miRNAs in Ctrl PM *vs* Ctrl comparison (**Figure 5A**; **Supplementary Table S5**), this pointed again to the special character of the response of EAE mice to PM_10_. In line with this idea, while the pathways described so far were largely shared across the four comparisons, several terms associated with fatty acid (FA) metabolism (*Biosynthesis of unsaturated fatty acids*, *Fatty acid metabolism*, *Fatty acid elongation*) were instead specifically enriched for the upregulated miRNA networks of EAE mice responding to PM (i.e. PM *vs* Ctrl PM and EAE PM *vs* EAE comparisons; **Figure 6A**; **Supplementary Table S6**).

### Gene-to-disease association of miRNA targets

**Figure 7:**
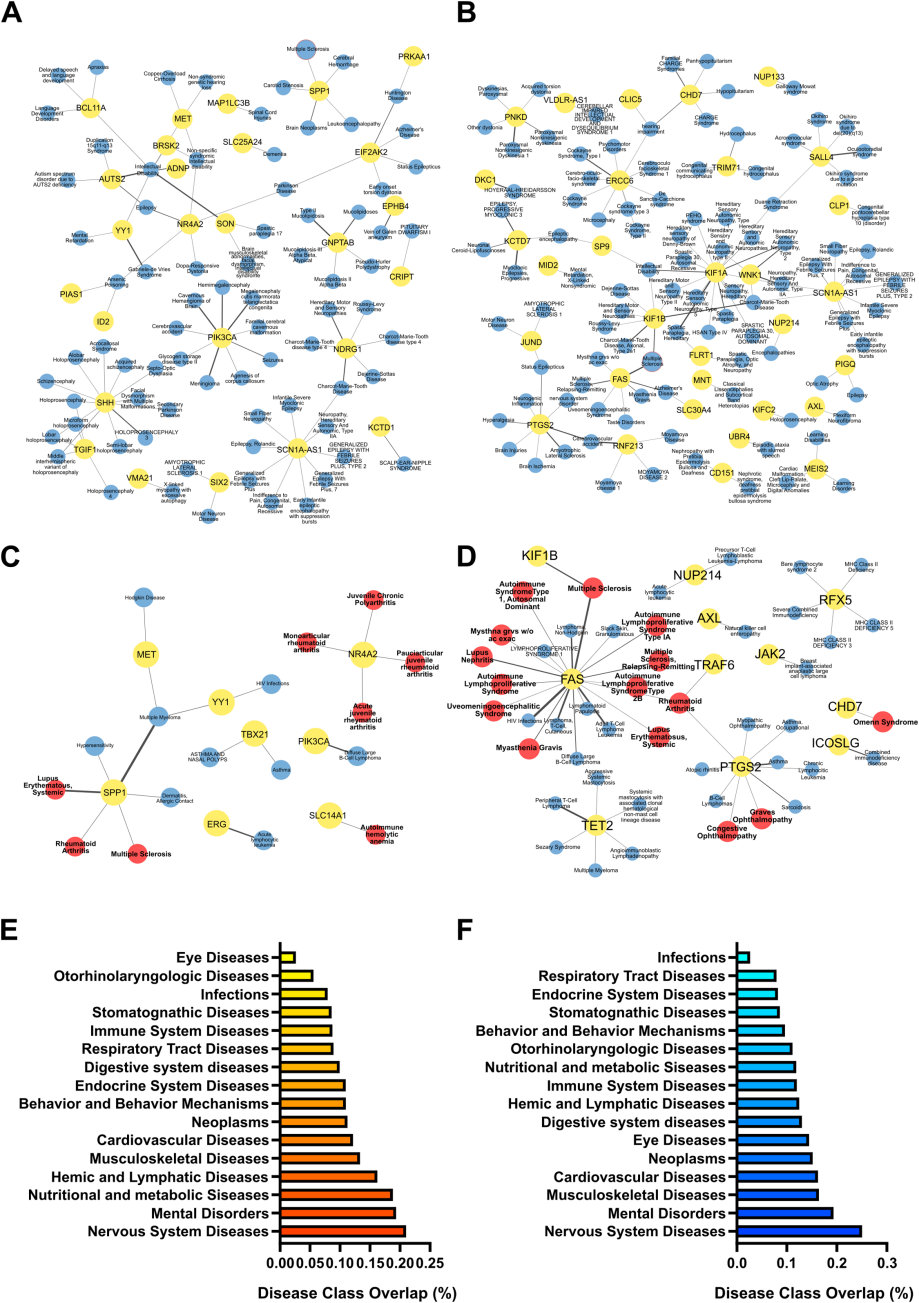
Pathological relevance of the selective response of EAE mice to PM_10_. Gene-to-disease association performed with DisGeNET performed for the target genes of miRNAs significantly upregulated **(A, C)** and downregulated **(B, D)** specifically in EAE PM vs Ctrl PM. **(A-D)** Networks of target genes (in yellow dots) associated with pathologies (in blue dots) in the DisGeNET disease class “Nervous Systems Diseases” **(A, B)** and “Immune System Diseases” **(C, D)**. Multiple Sclerosis (MS) node is highlighted with a red border in **(A, B)**. In the “Immune System Diseases” Network, Autoimmune diseases are represented by red dots. Thickness of links is proportional to the DisGeNET score, as a measure of quality/number of evidence supporting the gene-to-disease association. **(E, F)** Gene enrichment in disease classes found in the DisGeNET database. Bars represent the fraction of target genes over the entire number of genes listed in each disease class. Source data are provided in **Supplementary Table S7**.

To link EV-packaged miRNA dysregulation with possible pathological alterations elicited by PM exposure, we used the list of predicted target gene transcripts to perform a “gene-to-disease association” analysis. Again of relevance in the context of MS, and consistent with the KEGG pathway analysis, many of the targets of the miRNAs selectively deregulated in PM-exposed EAE *vs* PM-exposed healthy mice (i.e. EAE PM *vs* Ctrl PM; see **Supplementary Table S5**) are involved in nervous systems diseases and immune system diseases, and particularly in autoimmune diseases including MS (**Figures 7A–D**; **Supplementary Table S7**). Consistently, when assessing the fraction of gene transcripts targeted by miRNAs selectively deregulated in PM-exposed EAE mice over the entire number of genes listed in each of the disease classes included in the DisGeNET database, “Nervous System Diseases” was the most represented class, followed by Mental Disorders, Hemic and Lymphatic Diseases, Behavior and Behavior Mechanisms and Immune System Diseases (**Figures 7E, F**).

Overall, this analysis showed that gene transcripts targeted by miRNAs selectively altered in EAE mice exposed to PM_10_ were associated with nervous system disorders and with pathologies closely related to MS, suggesting that the plasmatic EV cargo may mediate the transfer of PM-induced damage to the CNS and synergize with the immune setting of the individual, possibly contributing to immune dysregulation in the CNS *via* yet unknown mechanisms.

## Discussion

With the aim of disclosing the biological bases of the higher vulnerability of immunologically-primed subjects to PM exposure, here we characterized plasmatic EVs and their miRNA cargo in healthy and presymptomatic EAE mice following a single exposure to high level PM_10_. We focused on PM_10_ because it has been consistently associated with MS-related outcomes in epidemiological studies (see above), and it includes finer fractions such as PM_2.5_. Results showed that the response of EAE mice to PM did not differ in terms of EV number and source, compared to that of healthy mice. Yet, remarkable differences existed in the identity of deregulated EV-associated miRNAs, which were predicted to target MS-relevant pathways and might contribute to disease worsening.

As regards EV abundance and features, PM_10_ exposure induced a significant decrease in plasmatic EV release in both healthy and EAE mice - accompanied by a moderate increase in size in PM_10_-exposed EAE mice - compared to unexposed individuals. These findings clash with what observed in humans - where short-term PM exposure has been associated with increases in plasmatic EVs (8, 15, 51), and may suggest a species-specific response, with possible change in EV release mechanisms in PM_10_-exposed EAE mice (42). Yet, EV numbers and size remained in the range of those reported in humans following PM exposure (8, 15) and EV size did not reach that of apoptotic bodies (42), thus not indicating higher toxicity of PM_10_ to murine cells.

As regards EV cargo, here we focused on EV-associated miRNAs as informative post-transcriptional markers, given their regulatory role and the interpretability of their dysregulation through known mRNA targets. In both healthy and EAE mice, the miRNA cargo of EVs released following PM exposure had a signature relevant for MS. However, deregulated miRNAs were distinct and the response of EAE mice appeared remarkably more “pathogenic” than that of healthy mice. Specifically, in Ctrl mice, exposure to PM_10_ led to a complex and “ambivalent” miRNA signature, including decreased “protective” miRNAs (e.g. miR-29a-5p; 52–55; miR-2134; 56), but also decreased proinflammatory miRNAs (e.g. miR-712-5p, 57) and increased anti-inflammatory miRNAs (e.g. miR-215-5p; 58, 59; miR146b-5p; 60, 61; miR-199a-3p; 62, 63). Of note, among miRNAs upregulated in healthy mice exposed to PM_10_, miR-146b-5p was formerly found upregulated in the blood and white matter tissue of MS patients and MS animal models (64), presenting a peak of expression in peripheral blood mononuclear cells (PBMCs) of treatment-naïve relapsing and remitting MS (RRMS) patients during the remission phase (65), in line with miR-146b-5p role as a molecular brake for T-cell B-cell crosstalk restraining autoimmunity (66). Similarly, miR-199a-3p expression in PBMCs was proposed to mediate the effects of disease-modifying therapies (DMTs) in RRMS patients (67), in line with its role in promoting immune tolerance (68).

Among these deregulated miRNAs, miR-215-5p and miR-199a-3p have been already reported as EV-packaged miRNAs in human serum (69) and mouse bronchoalveolar lavage fluid (70), respectively. Moreover, increased expression of miR-215-5p, miR-146b-5p and miR-199a-3p was detected in human and mouse lung epithelial cells upon exposure to PM_2.5_ and other pollutants (70–72).

All in all, these findings point to a prevalent compensatory/protective response elicited in healthy mice by short term exposure to PM_10_.

In contrast, in EAE mice, PM exposure resulted in a strong enrichment in miR-9-5p, whose level in the serum was correlated with clinical activity, brain atrophy and disability progression in MS patients (73–75). Such an association appears to rely on miR-9-5p role in microglia activation and neuroinflammation (76, 77), dendritic cell activation (78), Th17/Treg differentiation (79, 80), and oligodendroglia maturation (81). Notably, miR-9-5p was also one of the “hub” upregulated miRNAs highlighted by the network analysis in EAE mice exposed to PM_10_ (*vs* unexposed EAE mice and *vs* PM_10_ exposed healthy mice), corroborating the idea of its critical involvement in the response of EAE mice to PM_10_. In addition, EAE mice showed a strong decrease of anti-inflammatory miRNAs, such as miR-345-5p (82), miR-192-5p (83, 84) and miR-451a (85), compared to unexposed EAE mice. Of note, miR451a was formerly shown to play a wide array of immunoregulatory actions, by attenuating macrophage and dendritic cell responses (86), inhibiting T cell activation (87), suppressing NK cell activation and cytotoxicity (88) and regulating myeloid-derived suppressor cells differentiation in the context of autoimmune disease (85). In line with this, miR-451a was proposed to contribute to the remission phase of a number of autoimmune diseases, such as familial Mediterranean fever (89), rheumatoid arthritis (RA, 90) and systemic lupus erythematosus (SLE, 85, 91). Similar to miR-9-5p, miR-451a was also one of the “hub” downregulated miRNAs highlighted by the network analysis in EAE mice exposed to PM_10_ (*vs* unexposed EAE mice and *vs* PM_10_ exposed healthy mice), in line with a key role in EAE mouse response to PM_10_.

In healthy mice miRNAs deregulated after immunization and after PM_10_ exposure partly overlapped, suggesting a similar outcome in response to autoimmune and environmental triggers. In contrast, the response of EAE mice to PM_10_ was largely segregated from the response of healthy mice. Notably, while comparing the response of EAE and healthy mice to PM_10_ (i.e. EAE PM *vs* Ctrl PM), one of the top enriched miRNAs was miR-142-3p, which was shown to impact MS progression, severity, and therapeutic outcomes by orchestrating neuronal toxicity and excitotoxic synaptic alterations, (92–95), and possibly by regulating also Treg cell differentiation (96) and myelin repair (97). On the other hand, in addition to miR-451a, one of the top downregulated miRNAs in EAE PM *vs* Ctrl PM was the anti-inflammatory miR-25-3p (98), formerly found down-regulated in Treg cells of MS patients and proposed to alter Treg cell activity in MS by targeting TGF-β biological functions (99).

Overall, these findings indicate that the response to PM_10_ – in terms of deregulated plasma EV-associated miRNAs – is qualitatively different in mice primed to develop autoimmunity compared to healthy mice, and includes a repertoire of miRNAs that, if also deregulated in PM_10_-exposed MS patients, might contribute to key pathological aspects, including autoimmunity, CNS infiltration, neuroinflammation, neurodegeneration and myelin repair.

The special character of the response of EAE mice to PM_10_ and its relevance for MS were also highlighted by miRNA target prediction and network analysis, as well as by the analysis of the biological pathways potentially influenced by the detected miRNA deregulation. In general, the networks of upregulated miRNAs were remarkably complex, in line with the idea that upregulated miRNAs might have a broad biological impact, influencing a wide range of cellular processes. Pathways related to synapse function, neurotransmitter systems, nervous system, immune system and inflammation were significantly enriched for the networks of upregulated miRNAs in both healthy and EAE mice, whereas pathways related to immune system and inflammation emerged almost exclusively for the networks of downregulated miRNAs in EAE mice responding to PM_10_. Interestingly, among immune-related pathways, *Epstein Barr virus (EBV) infection* emerged as one of the most enriched pathways for downregulated miRNAs in PM-exposed EAE mice, suggesting that exposure to PM_10_ might result in the upregulation of target genes belonging to this category and might mimic the anti-EBV response in EAE mice. This finding is particularly intriguing as EBV infection is epidemiologically linked with the development of autoimmune diseases - including SLE, RA and MS - and multiple mechanisms associated with EBV infections (e.g. molecular mimicry, B cell reprogramming, crosstalk between EBV-infected B cells and T cells, etc.) are now considered causally implicated in autoimmunity and MS development (100, 101). Moreover, as an additional element of uniqueness of EAE mouse response to PM_10_, terms associated with FA metabolism (*Biosynthesis of unsaturated fatty acids*, *Fatty acid metabolism*, *Fatty acid elongation*) were specifically enriched for the upregulated miRNA networks of EAE PM *vs* Ctrl PM and EAE PM *vs* EAE comparisons. Of note, altered lipid profiles were reported in MS patients, with FA serological concentration reflecting disease activity and disability score, and a number of FA metabolism-related enzyme single nucleotide polymorphisms (SNPs) associated with MS incidence (102). Even before onset, MS patients display a unique FA serological profile and FA intake and metabolism are thought to contribute to MS susceptibility (102). Availability and profiles of FA species might alter the disease course by influencing immune cell function and specifically by modulating the polarization, differentiation, and cytokine production of T cells, which largely contribute to MS pathogenesis (103). Thus, a clear MS-relevant signature of the response of presymptomatic EAE mice to PM_10_ emerged, as also corroborated by “gene-to-disease association” analysis.

### Limitations of the study and future directions

Overall, our findings support the view that, in immunologically primed individuals - such as people with MS - exposure to PM_10_ can trigger a unique response through the delivery of EV-associated miRNAs, which can contribute to a worse manifestation or an unfavorable evolution of the pathology. Yet, our study has limitations that must be disclosed to correctly interpret our findings and to identify persisting open issues. Here, we combined exposure to PM_10_ with EAE, which is the closest animal model that approximates human MS pathogenesis by reflecting its autoimmune component (104). Yet, as our results point to a likely participation of exposure to PM_10_ in immune dysregulation, it is relevant to acknowledge that some differences exist in the innate and adaptive immune system of mice and humans (105), potentially limiting the translatability of our findings and calling for future investigations in MS patients. Moreover, besides the relatively small size of individuals used in this kind of *in vivo* experiments, here we used only female mice, as in most studies focusing on EAE. This choice relies on the well-known sexual dimorphism existing in MS, with women showing a higher susceptibility to the disease (106). Yet, disease progression appears worse in men (106) and mechanisms leading to EAE partially differ in female and male mice (107). Inclusion of male mice in future studies will validate the generalizability of our findings or, alternatively, unveil sex-specific aspects in the response to PM_10_ of immunologically primed individuals. Moreover, as regards the analysis of EV-packaged miRNAs, this study did not include EV subtype-specific miRNA profiling due to limited EV yield, and sample pooling - while necessary due to sample constraints and ethical (i.e. 3Rs) considerations - limited the assessment of inter-individual variability. Finally, our study investigated an acute (i.e. occurring within 6 hours) biological response triggered by a single exposure to PM_10_. While this was instrumental to investigate the short-term effect of the peaks of airborne PM_10_ which were associated with higher rates of hospital admissions for MS and MS relapses (22, 29), results obtained in this experimental setting might not reflect the biological responses elicited upon long-term or chronic exposure to PM_10_. Similarly, in our study PM_10_ dose was calculated to mimic daily peaks of PM_10_ concentration in the most polluted European areas (>250 μg/m^3^; 41). Whether exposure to higher PM_10_ concentrations - as detected in other geographical regions (108) - or to other risk factors epidemiologically associated with MS risk may converge on the same biological mechanisms unveiled by our study deserves further investigation.

## Data Availability

The datasets presented in this study can be found in online repositories. The names of the repository/repositories and accession number(s) can be found in the article/**Supplementary Material**.

## Supplementary material

Supplementary Figure 1 Global characterization of isolated EVs. **(A)** Western Blotting analyses on plasma EV lysates from 3 representative mice. WB for CD63 and CD9 shows an enrichment in EV samples. WB for markers of other cellular compartments (i.e., calnexin) shows no positivity in EV samples and a positive band in the mouse cerebral cortex extract (positive control). **(B)** Size profile of isolated EVs. Distribution of EV concentration across the 30–700 nm size range, as measured by NTA, for each experimental group (CTRL, CTRL PM, EAE, EAE PM). Each curve represents the mean relative abundance of EVs over replicate measurements, and the colored halo is defined by the standard error at each size.

Supplementary Figure 2 Agilent profiles of isolated miRNAs. Representative profiles of the miRNAs isolated from 2 mice, showing RNA integrity and suitability for subsequent analyses.
