# Supplementary material for: Short-term exposure to particulate matter triggers a selective alteration of plasma extracellular vesicle-packaged miRNAs in a mouse model of multiple sclerosis
Source: Front Immunol. 2025 Jul 3;16:1596935. doi: 10.3389/fimmu.2025.1596935 (PMC12267195; doi:10.3389/fimmu.2025.1596935)
Supplement: Supplementary file 1 [file Table1.docx]

# Supplementary Table 1 – MISEV2018 Guidelines Compliance

| **Section title** | **Required information according to MISEV2018** | **Mandatory requirement** | **Our approach** | **Compliance with MISEV2018 requirements** |
| --- | --- | --- | --- | --- |
| Nomenclature | The term extracellular vesicle (EV) can be used with demonstration of extracellular (no intact cells) and vesicular nature per these characterization and function | YES | The term extracellular vesicle (EV) was used consistently to describe vesicular particles of extracellular origin, isolated from plasma. | YES |
| 2a – Tissue culture medium | General cell characterization | YES | Not applicable | Not applicable |
| 2a – Tissue culture medium | Medium used before and during collection (additives, serum, etc.) | YES | Not applicable | Not applicable |
| 2a – Tissue culture medium | EV-depletion of additives | YES | Not applicable | Not applicable |
| 2a – Tissue culture medium | Culture vessel and volume | YES | Not applicable | Not applicable |
| 2a – Tissue culture medium | Number of cells and % live/dead | YES | Not applicable | Not applicable |
| 2a – Tissue culture medium | Conditioned medium harvest frequency | YES | Not applicable | Not applicable |
| 2b and 2c – Biofluids or tissues | Donor status (age, sex, diet, time of collection, etc.) | YES | Mice were matched by age and sex. Blood samples were collected between 1:00–2:00 PM and processed within 2 hours. | YES |
| 2b and 2c – Biofluids or tissues | Volume of biofluid or tissue collected | YES | plasma pools from six mice of each experimental condition; EV isolation from 1.5 mL plasma per pooled group. | YES |
| 2b and 2c – Biofluids or tissues | Pre-treatment to remove contaminants | YES | Sequential centrifugation (1000g to 3000g) to obtain platelet-free plasma and remove debris. | YES |
| 2b and 2c – Biofluids or tissues | Handling conditions (temperature/time) | YES | Samples processed within 2 hrs and maintained at 4°C. | YES |
| 2d – Storage and recovery | Storage/thawing of biofluids or tissues | YES | Not applicable (freshly processed samples) | Not applicable |
| 2d – Storage and recovery | Storage of EVs after isolation | YES | EVs were analyzed within 24 h by flow cytometry and NTA. | YES |
| 3 – EV separation and concentration | Centrifugation parameters | YES | Low-speed centrifugation followed by ultracentrifugation at 110,000g for 75 min, MLA-55 rotor. | YES |
| 3 – Density gradient | Gradient details and centrifugation | YES | Not applicable | Not applicable |
| 3 – Chromatography | Matrix and parameters | YES | Not applicable | Not applicable |
| 3 – Precipitation | Polymer-based methods | YES | Not applicable | Not applicable |
| 3 – Filtration | Membrane characteristics | YES | Not applicable | Not applicable |
| 3 – Antibody-based | Antibody details | YES | Not applicable | Not applicable |
| 4a – EV quantification | At least 2 methods | YES | NTA (NanoSight NS300) and high-resolution flow cytometry with CFSE labeling. | YES |
| 4a – EV quantification | Ratio of 2 quantification methods | YES | Not calculated explicitly | Not applicable |
| 4b – General characterization | 2 positive markers + 1 negative | YES | Western Blotting for CD63 and CD9 as positive EV markers, Calnexin as negative marker. | YES |
| 4c – Single EV characterization | EM images and/or non-image methods | YES | NTA and flow cytometry confirm single-EV level resolution. | YES |
| 5 – Functional studies | Dose-response | YES | Not applicable | Not applicable |
| 5 – Functional studies | Negative controls | YES | Not applicable | Not applicable |
| 5 – Functional studies | Activity of EV vs. depleted fluid | YES | Not applicable | Not applicable |
| 5 – Functional studies | Comparison of EV subtypes | YES | Not applicable | Not applicable |
| 5 – Functional studies | EV donor-recipient separation | YES | Not applicable | Not applicable |
| 6 – Reporting | Data deposition in public repositories | YES | Only targeted miRNA profiling performed; no omics data deposited. | Not applicable |
