# Supplementary material for: Short-term exposure to particulate matter triggers a selective alteration of plasma extracellular vesicle-packaged miRNAs in a mouse model of multiple sclerosis
Source: Front Immunol. 2025 Jul 3;16:1596935. doi: 10.3389/fimmu.2025.1596935 (PMC12267195; doi:10.3389/fimmu.2025.1596935)
Supplement: Supplementary file 2 [file Table2.docx]

| **Figure** | **Applied Test** | **n** | **P value** | **Statistics** | **Post hoc analyses** | **Post hoc results** |
| --- | --- | --- | --- | --- | --- | --- |
| 1b | (two way ANOVA) | n=4 | Immunization Effect: P= 0,0005  Exposure Effect: P<0,0001  Immunization x Exposure: P= 0,4744 | Immunization: F (1, 12) = 21,99  Exposure:  F (1, 12) = 52,28 Immunization x Exposure:  F (1, 12) = 0,5454 | Bonferroni’s Multiple Comparisons Test | CTRL Sal. vs CTRL PM: P= 0,0007  CTRL Sal. VS EAE Sal.: P=0,0142  CTRL PM vs EAE PM:  n.s.  EAE Sal. Vs EAE PM: P= 0,0037 |
| 1c | (two way ANOVA) | n=4 | Immunization Effect: P= 0,0020  Exposure Effect: P=0,0641  Immunization x Exposure: P= 0,1195 | Immunization: F (1, 12) = 15,50  Exposure:  F (1, 12) = 4,159  Immunization x Exposure:  F (1, 12) = 2,810 | Bonferroni’s Multiple Comparisons Test | CTRL Sal. vs CTRL PM: n.s.  CTRL Sal. VS EAE Sal.: n.s.  CTRL PM vs EAE PM:  P= 0,0112  EAE Sal. Vs EAE PM: n.s. |
| 1d (CD14+) | (two way ANOVA) | n=4 | Immunization Effect: P= 0,0027  Exposure Effect: P= 0,0695  Immunization x Exposure: P= 0,3804 | Immunization: F (1, 12) = 14,10  Exposure:  F (1, 12) = 3,973  Immunization x Exposure:  F (1, 12) = 0,8293 | Bonferroni’s Multiple Comparisons Test | CTRL Sal. vs CTRL PM: n.s.  CTRL Sal. VS EAE Sal.: P= 0,0381  CTRL PM vs EAE PM:  n.s.  EAE Sal. Vs EAE PM: n.s. |
| 1d (CD41+) | (two way ANOVA) | n=4 | Immunization Effect: P= 0,2144  Exposure Effect: P= 0,3182  Immunization x Exposure: P= 0,3228 | Immunization: F (1, 12) = 1,718  Exposure:  F (1, 12) = 1,084  Immunization x Exposure:  F (1, 12) = 1,063 | Bonferroni’s Multiple Comparisons Test | CTRL Sal. vs CTRL PM: n.s.  CTRL Sal. VS EAE Sal.: n.s.  CTRL PM vs EAE PM:  n.s.  EAE Sal. Vs EAE PM: n.s. |
| 1d (CD25+) | (two way ANOVA)) | n=4 | Immunization Effect: P= 0,0041  Exposure Effect: P= 0,1484  Immunization x Exposure: P= 0,1980 | Immunization: F (1, 12) = 12,49  Exposure:  F (1, 12) = 2,386  Immunization x Exposure:  F (1, 12) = 1,857 | Bonferroni’s Multiple Comparisons Test | CTRL Sal. vs CTRL PM: n.s.  CTRL Sal. VS EAE Sal.: n.s.  CTRL PM vs EAE PM:  P= 0,0282  EAE Sal. Vs EAE PM: n.s. |
