# Supplementary figures and images for: Short-term exposure to particulate matter triggers a selective alteration of plasma extracellular vesicle-packaged miRNAs in a mouse model of multiple sclerosis
Source: Front Immunol. 2025 Jul 3;16:1596935. doi: 10.3389/fimmu.2025.1596935 (PMC12267195; doi:10.3389/fimmu.2025.1596935)

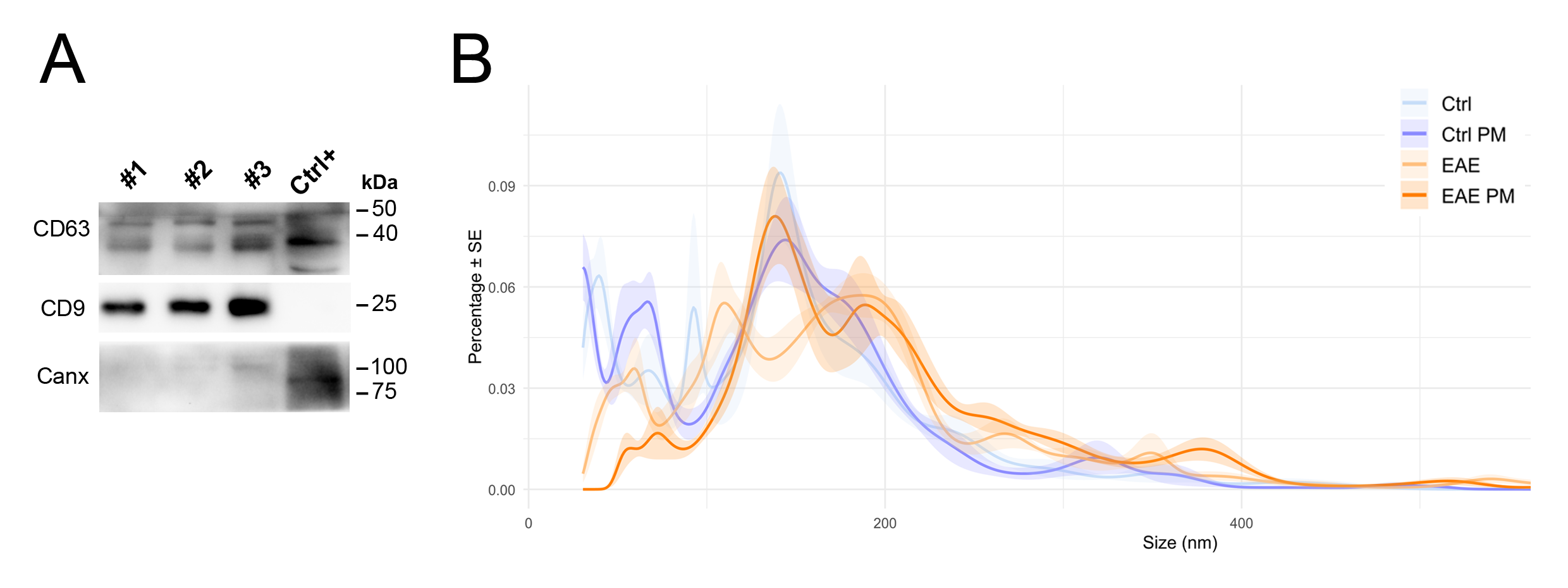

Supplement: Supplementary Figure 1 — Global characterization of isolated EVs. (A) Western Blotting analyses on plasma EV lysates from 3 representative mice. WB for CD63 and CD9 shows an enrichment in EV samples. WB for markers of other cellular compartments (i.e., calnexin) shows no positivity in EV samples and a positive band in the mouse cerebral cortex extract (positive control). (B) Size profile of isolated EVs. Distribution of EV concentration across the 30–700 nm size range, as measured by NTA, for each experimental group (CTRL, CTRL PM, EAE, EAE PM). Each curve represents the mean relative abundance of EVs over replicate measurements, and the colored halo is defined by the standard error at each size. [file Image1.tif]

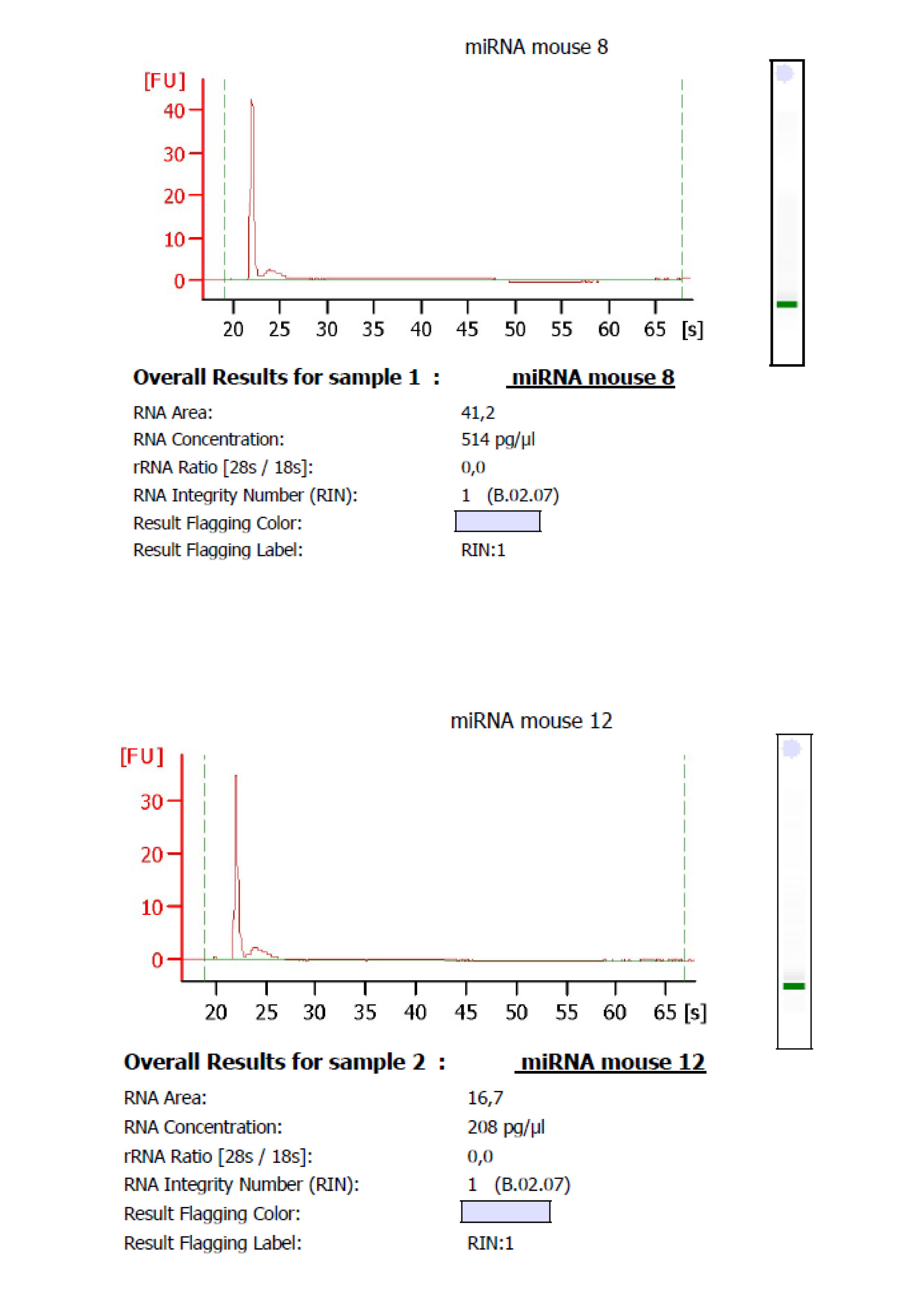

Supplement: Supplementary Figure 2 — Agilent profiles of isolated miRNAs. Representative profiles of the miRNAs isolated from 2 mice, showing RNA integrity and suitability for subsequent analyses. [file Image2.tif]
